# hnRNP A1-mediated translational regulation of the G quadruplex-containing RON receptor tyrosine kinase mRNA linked to tumor progression

**DOI:** 10.18632/oncotarget.7589

**Published:** 2016-02-22

**Authors:** Anne Cammas, Magali Lacroix-Triki, Sandra Pierredon, Morgane Le Bras, Jason S. Iacovoni, Marie-Paule Teulade-Fichou, Gilles Favre, Henri Roché, Thomas Filleron, Stefania Millevoi, Stéphan Vagner

**Affiliations:** ^1^ INSERM UMR 1037, Centre de Recherches en Cancérologie de Toulouse, Toulouse, France; ^2^ Université Toulouse III Paul Sabatier, Toulouse, France; ^3^ Institut Claudius Regaud, Toulouse, France; ^4^ Institut Curie, PSL Research University, CNRS UMR 176, Orsay, France; ^5^ Institut Curie, PSL Research University, CNRS UMR 3348, Orsay, France; ^6^ Université Paris Sud, Université Paris-Saclay, CNRS UMR 176, Orsay, France; ^7^ Université Paris Sud, Université Paris-Saclay, CNRS UMR 3348, Orsay, France; ^8^ Equipe Labellisée Ligue Contre le Cancer, Paris, France

**Keywords:** breast cancer, metastasis, RNA-binding protein, RON, translation

## Abstract

The expression and role of RNA binding proteins (RBPs) controlling mRNA translation during tumor progression remains largely uncharacterized. Analysis by immunohistochemistry of the expression of hnRNP A1, hnRNPH, RBM9/FOX2, SRSF1/ASF/SF2, SRSF2/SC35, SRSF3/SRp20, SRSF7/9G8 in breast tumors shows that the expression of hnRNP A1, but not the other tested RBPs, is associated with metastatic relapse. Strikingly, hnRNP A1, a nuclear splicing regulator, is also present in the cytoplasm of tumor cells of a subset of patients displaying exceedingly worse prognosis. Expression of a cytoplasmic mutant of hnRNP A1 leads to increased translation of the mRNA encoding the tyrosine kinase receptor RON/MTS1R, known for its function in tumor dissemination, and increases cell migration *in vitro*. hnRNP A1 directly binds to the 5′ untranslated region of the *RON* mRNA and activates its translation through G-quadruplex RNA secondary structures. The correlation between hnRNP A1 and RON tumoral expression suggests that these findings hold clinical relevance.

## INTRODUCTION

The regulation of protein synthesis, established to be a crucial component of cancer cell survival and transformation [1], is emerging to play a role in cell invasiveness and metastasis as well. Recently, translation up-regulation of messenger RNAs (mRNAs) encoding proteins that increase cell invasiveness was demonstrated to direct cancer invasion and metastasis downstream of oncogenic mTOR signalling [2]. Although this regulation was shown to be linked to specific *cis*-acting elements found in the 5′ untranslated region (5′UTR) of translationally regulated mRNAs, no *trans*-acting factors, such as specific RNA binding proteins (RBPs) known to regulate translation were demonstrated to be involved in this regulation. However, Y-box binding protein-1 (YB-1), a DNA/RNA binding protein known to shuttle between the nucleus and cytoplasm of cells, was among the genes found in the pro-invasion signature [2]. YB-1 was previously shown to act as a translation factor that controls expression of a larger set of genes involved in cancer cell invasion [3]. Direct binding of YB-1 to the translationally-regulated mRNAs was not investigated. The best evidence for the translational role of an RBP with a specific interaction with an mRNA encoding a protein involved in cell invasiveness exists for the fragile X mental retardation protein FMRP [4] and for hnRNP E1, a member of the heterogeneous nuclear ribonucleoprotein (hnRNP) family of RBPs [5].

To find out whether other RBPs regulate translation initiation of specific mRNAs during tumor progression, we first analysed by immunohistochemistry (IHC) the expression of several RBPs (hnRNP A1, hnRNP H, RBM9/FOX2, SRSF1/ASF/SF2, SRSF2/SC35, SRSF3/SRp20, SRSF7/9G8) in breast cancers. These RBPs were chosen based on their previously demonstrated translational activities [6–9]. Here, we describe that expression of hnRNP A1, but not of the other tested RBPs, is associated with metastatic relapse in breast cancer. We also show that hnRNP A1 binds to G-quadruplex (G4) RNA elements in the *RON/MTS1R* 5′UTR. *RON* encodes a tyrosine kinase receptor known for its function in tumor dissemination and a correlation exists between protein levels of hnRNP A1 and RON in breast tumors.

## RESULTS

### RBP expression in breast cancer

**Figure 1:**
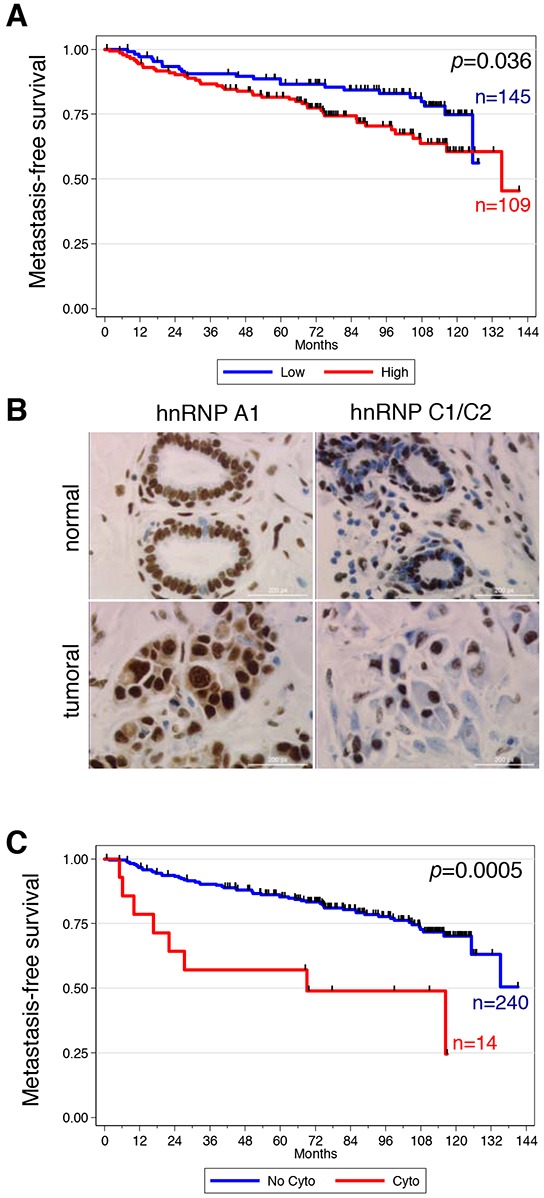
High expression and cytoplasmic localization of hnRNP A1 are associated with metastatic relapse in patients with invasive breast cancer **A.** Kaplan-Meier analysis showing that high hnRNP A1 expression is significantly associated with lower distant metastasis-free survival. **B.** Immunohistochemistry performed in the normal and tumor breast samples from the same patient. **C.** Kaplan-Meier analysis showing that hnRNP A1 cytoplasmic localization is significantly associated with lower distant metastasis-free survival.

Expression of RBPs was measured by immunohistochemistry (IHC) in a collection of 277 breast cancer specimens (Supplementary Table S1). Due to tissue loss in the tissue microarray (TMA), immunostainings (Supplementary Figure S1) were interpretable in 249 to 256 cases depending on the RBP (Supplementary Table S2). Analysis of the correlation between RBP expression and clinicopathological features is shown in Supplementary Table S3. We found a positive correlation between histological grade and the expression of hnRNP H (*p* = 0.048) and a negative correlation between histological grade and expression of SRSF3 (*p* = 0.03). We found a positive correlation between lymph node metastasis and expression of hnRNP A1 (*p* < 0.01) or SRSF7 (*p* < 0.01) and a negative correlation between lymph node metastasis and the expression of hnRNP H (*p* = 0.03) or SRSF3 (*p* < 0.01). Associations were also found between estrogen receptor (ER) status and expression of SRSF3 (*p* < 0.01) and RBM9 (*p* < 0.01) as well as between HER2 status and expression of SRSF3 (*p* = 0.03) and SRSF7 (*p* = 0.017). No associations were observed between expression of hnRNP A1, SRSF1 or SRSF2 and histological grade, molecular subtype or the status of either hormonal receptors or HER2. Kaplan-Meier analysis showed that hnRNP A1 expression correlated with clinical outcome (Supplementary Table S4). Indeed, patients with a high level of hnRNP A1 expression had a reduced distant metastasis-free survival, with a 10-year survival rate of 60% in the hnRNP A1^high^ group *vs* 74% in the hnRNP A1^low^ group (*p* = 0.036, Figure 1A). As expected given the association between hnRNP A1 expression and lymph node status, hnRNP A1 prognostic correlation was not retained on multivariate analysis (data not shown). Of note, we controlled the validity of hnRNP A1 antibody for IHC experiments; three breast cancer tumor samples displaying different levels of hnRNP A1 expression as assessed by IHC were also analyzed by western blot experiment on frozen matched tumor sample, and showed the same level of protein expression as determined by IHC (Supplementary Figure S2).

### Subcellular localization of hnRNP A1 in breast cancer

hnRNP A1, although predominantly nuclear and involved in the regulation of alternative pre-mRNA splicing, is able to shuttle between the nucleus and the cytoplasm [10] where it binds and regulates translation of several mRNAs [7, 10–12]. We therefore investigated the subcellular localization of hnRNP A1 in breast cancers. Out of the 254 interpretable study patients, we observed cytoplasmic staining in 14 breast carcinoma specimens, with exclusively nuclear staining in all matched normal breast tissues (Figure 1B). This finding was specific for hnRNP A1 and not observed on the same samples for hnRNP C1/C2 (Figure 1B), known to shuttle between the nucleus and cytoplasm. Among these 14 cases with cytoplasmic localization, 12 displayed a high level of hnRNP A1 expression, with an IRS score > 9 (Supplementary Table S5). Consistent with this observation, we observed some degree of overlap in the clinicopathological characteristics of tumors displaying high hnRNP A1 expression and cytoplasmic localization (Supplementary Tables S5 and S6). However, tumors displaying cytoplasmic localization of hnRNP A1 were more likely to be of larger size (*p* = 0.0260, Supplementary Table S5). Most strikingly, the presence of cytoplasmic hnRNP A1 expression correlated extremely well with metastatic relapse (10-yr metastasis-free survival of 24.5% in the cytoplasmic group *vs* 70.1%, *p* = 0.0005, Figure 1C) and poor outcome (10-yr overall survival of 31.2% in the cytoplasmic group *vs* 70.4%, *p* = 0.0087).

### hnRNP A1 binds to the 5′UTR of the mRNA encoding the RON receptor tyrosine kinase and regulates its expression

Since we are particularly interested in defining RBPs regulating the translation of mRNAs involved in metastatic process, we investigated hnRNP A1 translational targets. hnRNP A1 is known to regulate the translation of mRNA targets, either positively or negatively, by directly interacting with target 5′UTRs [7, 10–12]. hnRNP A1 recognizes the UAGGGA/U RNA sequence with high affinity [13]. A bioinformatics search allowed us to find 120 mRNAs containing at least one UAGGGA/U sequence in their 5′UTR and encoding a protein involved in cell migration and breast cancer. Strikingly, among these 120 encoding mRNAs, 19 also possess a G4 motif in their 5′UTR (Supplementary Table S7). G4 RNA structures, present in the 5′UTR of mRNAs, are known to generally inhibit translation [14]. mRNAs containing a G4 in their 5′UTR encode a number of oncogenes and are regulated at the translation level by eIF4A, a general translation initiation factor [15].

**Figure 2:**
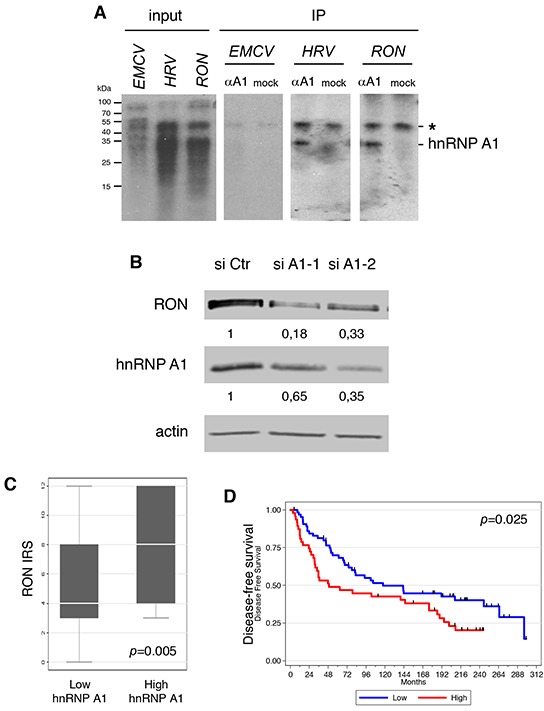
hnRNP A1 binds to the 5′UTR of the *RON* mRNA and increases the expression of RON **A.** UV cross-linking of cell extracts with ^32^P-labeled *in vitro* transcribed RNAs corresponding to the 5′UTR of the *RON* mRNA or to the *EMCV* and *HRV* IRES. The positions of protein molecular-weight markers in kilodaltons (kDa) are indicated on the left-hand side of the gels. Immunoprecipitation (IP) of crosslinked RNA-protein complexes was performed with (αA1) or without (mock) the 4B10 antibody directed against hnRNP A1. (*) indicates a nonspecific band. **B.** Western blot analysis of RON and hnRNP A1 expression in the hnRNP A1-depleted (Two different siRNAs: si-A1-1 and siA1-2) MDA-MB-231 cells using antibodies against RON, hnRNP A1 and actin. **C.** Boxplot showing the statistically significant positive correlation between RON expression and hnRNP A1 expression in breast cancer specimens. **D.** Kaplan Meier analysis showing that high RON expression is significantly associated with lower distant metastasis-free survival in breast cancer patients.

Beyond the role of a general translation initiation factor in G4-dependent translation, we wanted to investigate the possible role of a specific RBP in G4-dependent translation. Among the 19 mRNAs, we focused on *RON* (Recepteur d'Origine Nantais; a name based on the French city of its discovery), which encodes macrophage stimulating 1 receptor (MST1R), a member of the Met family of receptor tyrosine kinases [16], for the following reasons. First, RON activation leads to cellular growth, motility, and invasion [17]. Second, RON overexpression in a variety of human cancers often correlates with metastasis and poor outcome [18]. We investigated the interaction between hnRNP A1 and the *RON* 5′UTR using a UV crosslinking/immunoprecipitation assay. As expected, hnRNP A1 bound to one of its known targets, the human rhinovirus (HRV) IRES (Figure 2A). hnRNP A1 directly interacted with the *RON* 5′UTR and the known target from the human rhinovirus (HRV) IRES [7, 10] but not to the encephalomyocarditis virus (EMCV) IRES (Figure 2A). We next evaluated the relationship between hnRNP A1 and RON expression. Western blotting showed that siRNA-mediated depletion of hnRNP A1 in the invasive breast cancer cell line MDA-MB-231 cells decreased RON protein levels (Figure 2B) but had little effect on *RON* mRNA levels (Supplementary Figure S3A). Interestingly, we found a positive correlation between the expression of hnRNP A1 and RON in the breast tumor collection (Figure 2C), in which RON also significantly correlated with clinical outcome. Indeed, Kaplan-Meier analysis showed that patients with a high level of RON expression had significantly reduced distant disease-free survival, with a 10-year survival rate of 42.5 % in the RON^high^ group *vs* 49.7 % in the RON^low^ group (*p* = 0.025, Figure 2D). Altogether these data indicate that high expression of hnRNP A1, an RBP that binds to the 5′UTR of the *RON* mRNA, is associated with overexpression of RON.

### An hnRNP A1 mutant with a cytoplasmic localization, increases the expression of RON and cell migration

**Figure 3:**
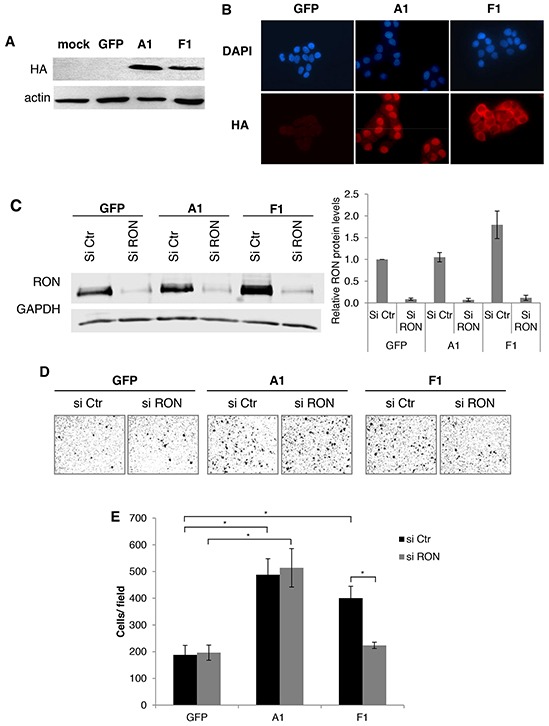
The cytoplasmic mutant of hnRNP A1 increases *RON* expression and cell migration **A.** Western blot analysis of the expression of hnRNP A1 (A1), the hnRNP A1 cytoplasmic mutant (F1) or the GFP control (GFP) in transduced T47D cells using antibodies against the HA tag and actin. **B.** Immunofluorescence assay on T47D transduced with the cytoplasmic mutant of hnRNP A1, hnRNP A1 and GFP by using anti-HA-Cy5/DAPI staining. **C.** Western blot analysis of the expression of RON in the T47D cell line using antibodies against RON and GAPDH. The basal levels of RON normalized to GAPDH in the control condition were arbitrarily set at 1.0 and the fold change of each condition was plottes +/− S.E.M. from three independent experiments. **D.** Representative images of migration assays (Boyden chambers) performed with the same cell lines and treatments described in C. **E.** Migration assays (Boyden chambers) performed with the same cell lines and treatments described in C. (*p < 0.05).

To specifically analyze the contribution of the cytoplasmic localization of hnRNP A1 in RON expression and cell migration, we ectopically expressed a cytoplasmic mutant of hnRNP A1 (called F1 [19]) (Figure 3A and 3B). The expression of F1, but not hnRNP A1, increased RON protein levels (Figure 3C) but had no effect on *RON* mRNA levels (Supplementary Figure S3B). Importantly, while both F1 and hnRNP A1 increased cell migration, siRNA-mediated depletion of RON (Figure 3C) reduced the effect of F1 on cell migration but had no effect on hnRNP A1-mediated cell migration (Figure 3D and 3E). The observed increase in cell migration was not due to increased cell proliferation (Supplementary Figure S4). These data show that part of the effect of F1 on cell migration is mediated by its effect on RON expression.

### F1 regulates the translation of the *RON* mRNA

**Figure 4:**
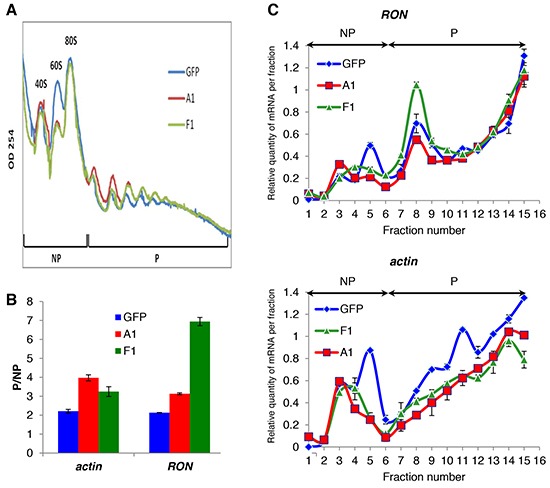
The cytoplasmic mutant of hnRNP A1 increases *RON* mRNA translation **A.** Polysome profiles of T47D GFP or a stably transduced T47D expressing the HA-tagged F1 or hnRNP A1 fractionated through sucrose gradients (15-50% sucrose). Absorbance at wavelength 254 nm was measured in order to determine the profile of polysome distribution. RNA extracts were prepared from the non-polysome (NP) and polysome (P) fractions or from each fraction. **B.** qRT-PCR was performed on NP and P fractions using specific primers for *actin* and *RON* mRNAs. The ratio (mRNA P/mRNA NP) in the indicated cell lines was plotted relatively to the T47D cells +/− S.E.M. from three independent experiments. **C.** The abundance of the *RON* and *actin* transcripts in 16 equal fractions (volume) derived from A. were quantified by qRT-PCR. The relative amount of each mRNA in each fraction was calculated. The abundance of the *RON* transcripts in the different “inputs” (RNAs extracted from cytoplasmic extracts that are loaded on the sucrose gradient) can be found in Supplementary Figure S3C.

To next assess whether F1 would regulate the translation of the *RON* mRNA, polysome gradients were prepared from cytoplasmic extracts of F1-transfected, hnRNP A1-transfected or control T47D cells (Figure 4A). The analysis of the polysomal mRNA distribution by quantitative reverse transcription PCR (qRT-PCR) showed that the expression of F1 induced *RON* to shift from the non-polysomic to the polysomic fractions of the gradient, thereby revealing an increase in *RON* mRNA translation (Figure 4B and 4C). This effect was specific as the recruitment of *actin* mRNA was not affected in the same way (Figure 4B and 4C). The expression of hnRNP A1 had no effect on the translation of the *RON* mRNA. Altogether, we concluded that the forced ectopic expression of a cytoplasmic mutant of hnRNP A1 leads to increased translation of *RON* mRNA and increased cancer cell migration.

### hnRNP A1-mediated regulation of *RON* mRNA requires G4 RNA structures found in the *RON* 5′UTR

**Figure 5:**
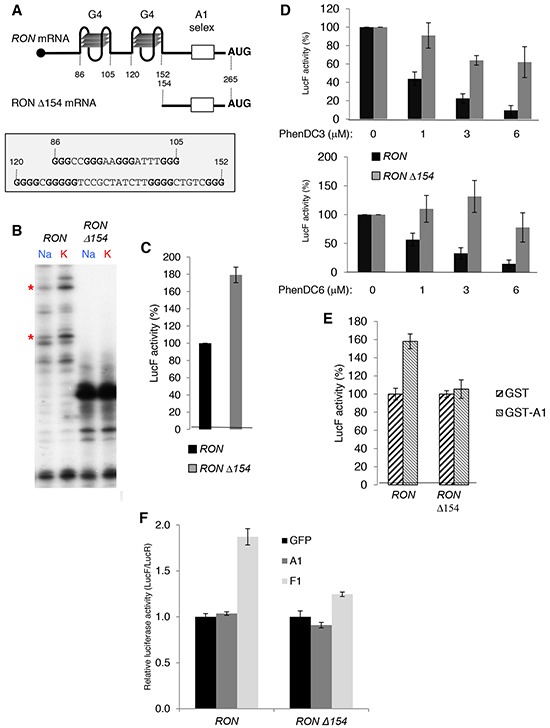
G4 RNA structures in the 5′UTR of the *RON* mRNA are required for hnRNP A1-mediated translation activation **A.** Schematic diagram of *RON* or *RON Δ154* 5′UTRs. Two G4s predicted by the QGRS Mapper software and the hnRNP A1 high affinity binding site (A1 selex) are indicated. **B.** Cation-dependent termination of reverse transcription for *RON* and *RON Δ154* RNA. Strong pauses of reverse transcriptase are indicated by asterisks. **C.** *In vitro* translation assays of firefly reporters containing either *RON* or *RON Δ154* 5′UTRs upstream of the luciferase firefly gene in rabbit reticulocyte lysate (RRL). **D.** *In vitro* translation assays of firefly reporters (see C) with increasing amounts of the PhenDC3 (DC3) or PhenDC6 (DC6) G4 ligands. **E.** *In vitro* translation assays of firefly reporters (see C) supplemented with 50 ng of recombinant GST- and GST-hnRNP A1. GST controls are fixed at 100%. F. LucF/LucR ratio for the indicated cells transfected with a capped polyadenylated transcript containing the LucR ORF (for normalization) and capped/polyadenylated transcripts containing either the *RON* 5′UTR or the *RON Δ154* 5′UTR upstream of the LucF ORF.

To determine how hnRNP A1 could regulate the translation of *RON* mRNA, we first focused on two predicted G4 RNA structures present in the *RON* 5′UTR between positions +85 and +154 (Figure 5A). We intended to confirm the presence of the two G4s by analyzing the influence of the nature of the cation on the pauses of reverse transcription since planar layers of G-quartets are stabilized preferentially by K+ in comparison to Na+. We observed two pauses in presence of KCl that partly disappeared in presence of NaCl suggesting the presence of G4 structures (Figure 5B). In order to determine the function of these RNA structures on *RON* translation, we constructed plasmids to *in vitro* synthesize reporter RNAs containing either the *RON 5*′UTR (WT) or the deleted mutant (*Δ154)*, in which the two G4s are deleted) upstream of the luciferase *Firefly* (LucF) open reading frame (ORF). *In vitro* translation showed that deleting the G4 region in the Δ154 RNA led to an increase in translation (Figure 5C). The bisquinolinium derivatives PhenDC3 and PhenDC6, which are described to stabilize RNA G4s [20–22], inhibited translation of *RON* 5′UTR-containing RNA in a dose dependent manner and did not significantly affect translation of *Δ154* RNA (Figure 5D), supporting the conclusion that the G4s located in the first 154 nucleotides of the *RON* 5′UTR were responsible for the inhibition of translation. The addition of recombinant hnRNP A1 increased the translation of *RON* 5′UTR-containing RNA but had no effect on the translation of *Δ154* RNA (Figure 5E). Furthermore, the normalized LucF activity driven by a transfected reporter transcript containing the *RON* 5′UTR upstream of the LucF ORF was higher in F1-expressing cells as compared to A1-expressing or mock-transfected T47D cells (Figure 5F). This effect was not observed for the *Δ154 RON* 5′UTR construct (Figure 5F). Altogether, these data indicate that binding of hnRNP A1 to the *RON* 5′UTR activates the translation of *RON* mRNA in a G4-dependent manner.

## DISCUSSION

In summary, among the 7 RBPs that were tested in this study, we found that hnRNP A1 was overexpressed in breast tumors of patients with a poor outcome. hnRNP A1 has been reported to be involved in tumor dissemination [23, 24]. However, the contribution of the subcellular localization of this shuttling protein in tumor progression has not been investigated. The use of an hnRNP A1 mutant that localizes predominantly to the cytoplasm allowed us to demonstrate that cytoplasmic hnRNP A1 contributes to cell migration by regulating gene expression at the translation level. The involvement of RON in the effect of cytoplasmic hnRNP A1 on cell migration is supported by (i) the increase in *RON* mRNA translation leading to increased *RON* protein levels and (ii) the fact that siRNA-mediated depletion of RON prevents the effect of F1 on cell migration. The increased cell migration observed in A1-overexpressing cells is however not dependent on RON. It is therefore possible that, in the nucleus, hnRNP A1 regulates alternative splicing of several genes involved in cell migration/invasion.

However, besides the hnRNP A1-mediated translation regulation of the *RON* mRNA described here, the *RON* gene is also regulated at the splicing level by hnRNP A1 [23]. It therefore seems that multiple hnRNP A1-dependent mechanisms may together be responsible for regulating RON expression during tumor progression. This coordination should be considered in further studies examining the role of hnRNP A1 in physiological/pathological processes. This is also certainly not restricted to the specific case of hnRNP A1. Indeed, this dual function whereby hnRNP A1 acts as both a splicing and a translational regulator for the same gene is reminiscent of a recent study showing that another RBP, CPEB1, mediates nuclear alternative 3′UTR processing in coordination with cytoplasmic translational regulation [25].

Computational analysis revealed that G4-forming motifs are overrepresented in the 5′UTR of mRNAs [26, 27]. G4-forming motifs were also found in mRNAs whose translation is regulated by silvestrol, an inhibitor of the eIF4A general translation initiation factor [15]. The group of genes encoding eIF4A-dependent mRNAs included many well-known oncogenes, such as B cell lymphoma 2 (BCL2), c-Myc (MYC) and notch 1 (NOTCH1), neuroblastoma Ras viral (v-Ras) oncogene (NRAS) and VEGF.[15] mRNAs containing G4s in their 5′UTR are particularly interesting in light of recent findings showing that they may be specifically targeted by eIF4A inhibitors [15], which also show promising anticancer activities [28]. Here, we identified a novel mechanism of translation regulation for mRNAs containing a G4 involving the specific binding of an RBP to the 5′UTR. Future work will aim to identify the full repertoire of mRNAs regulated at the translational level by hnRNP A1 during tumor progression.

## MATERIALS AND METHODS

### Plasmid construction

EMCV IRES was amplified by PCR using the pCREL plasmid as a template (Cammas et al., 2007) and the following primers : forward 5′-CGG GGA TCC ACT AGA ATC GAT CCC GCG A-3′ (*BamHI* restriction site is underlined) and reverse 5′-CGG CCA TGG TAT CAT CGT GTT TTT CAA AGG A-3′ (*NcoI* restriction site is underlined). The hnRNP A1 cytoplasmic mutant F1 was amplified by PCR using the following primers: forward 5′-CGG *CCA TGG* ATG GGT **TAC CCA TAC GAT GTT CCA GAT TAC GCT** TCT AAG TCA GAG TCT CCT AAA-3′ (*NcoI* restriction site is in italics, the HA tag sequence is in bold) and reverse-5′-CCG CTC GAG TTA AAA TCT TCT GCC GTC GCC ATA **ATC GTC ATC GTC ATC GTC** ACC GCC ATA GCC ACC TTG GTT TCG TGG-3′ (*XhoI* restriction site is underlined, the nucleotides modified to generate the mutant F1 are in bold). The trimolecular ligation was performed between the EMCV IRES PCR fragment digested by *BamHI* and *NcoI*, the F1 PCR fragment digested by *NcoI* and *XhoI* and the pcDNATM6.2-GW/EmGFP-LacZ plasmid from the BLOCK-iT™ Pol II miR RNAi ExpressionVector Kits (Invitrogen cat. K4935-00) digested by *BamH I* and *XhoI*. The lentiviral vector pLenti6/V5-EmGFP-IRES EMCV- HA F1 and the control pLenti6/V5- EmGFP- LacZ were then generated using the BLOCK-iT™ Pol II miR RNAi Expression system (Invitrogen cat. K4937-00) according to the manufacturer's protocol.

### Cases selection and tissue microarray (TMA) construction

The tumors selected for this study were retrieved from our institution's archives. Patient characteristics and clinicopathological features are described in Supplementary Table S1. Collection 1 consists of a series of 277 consecutive breast cancer patients treated by surgery with or without adjuvant treatment (chemotherapy, endocrine or radiation therapy) between 1996 and 1998. In 120/277 (43%) cases, systemic treatment consisted of an anthracycline-based chemotherapy, which was systematically given to patients with node-positive disease. Collection 2 is a long follow-up series of 113 breast cancer patients treated at our institution for invasive breast cancer between 1980 and 1983, who did not receive any adjuvant chemotherapy. Endocrine therapy was delivered in 107/277 (38.6%, Collection 1) and 62/113 (54.9%, Collection 2) cases. All patients treated by breast conservative surgery also received radiation therapy. This study was approved by local ethical committees.

Case selection for the TMA was based on the availability of archival paraffin blocks with sufficient tumor material for analysis. All tumor specimens included in this study were fixed in Bouin's solution. Elston-Ellis histological grade and nodal status. For the TMA construction, 600-μm diameter cores of histologically confirmed invasive breast carcinomas were extracted from the original (donor) paraffin blocks and re-embedded into a gridded recipient paraffin block using a Beecher Inc. tissue arrayer (Alphelys, Plaisir, France). For each case, 3 tumor cores and 1 normal breast core were taken from the original block. Control tissue cores from normal lymph node and placenta were also included in each recipient block. Medical reports of all cases were reviewed and clinical data (*i.e*. date and type of relapse, treatment, date and cause of death) were recorded.

### Immunohistochemistry

Immunohistochemistry (IHC) was performed on 4-μm-thick routinely processed paraffin TMA sections using a Techmate Horizon (Dako, Glostrup, Denmark) slide processor. A prior antigen retrieval heating-based technique was used for all immunostainings except for hnRNP A1 and hnRNP C1/C2. The primary antibodies used were directed against Estrogen Receptor a (ER) (clone 6F11, Novocastra, Newcastle, UK, dilution 1:50), Progesterone Receptor (PR) (clone PgR636, Dako, dilution 1:50), HER2 (polyclonal A0485, Dako, dilution 1:500), Cytokeratins (CK) 5/6 (clone D5/16B4, Dako, dilution 1:25), EGFR (clone 2-18C9, EGFR pharmDX™ kit, Dako, ready-to-use), hnRNP C1/C2 (clone 4F4, Abcam, Cambridge, UK, 1:3000), hnRNP A1 (clone 4B10, Abcam, dilution 1:6000), RBM9 (clone ab57154, Abcam, dilution 1:300), RON (clone 52927, Abcam, dilution 1:100), SRSF1 (polyclonal, kindly provided by Dr James Stevenin, dilution 1:6000), SRSF2 (clone SC-35, Abcam, dilution 1:1500), SRSF3 (clone 7B4A12, Invitrogen, Carlsbad, CA, dilution 1:300) and SRSF7 (clone 9G8, kindly provided by Dr James Stevenin, dilution 1:10). After inhibition of endogenous peroxidase (peroxidase blocking solution, Dako), slides were incubated with antibodies for 1h, followed by incubation with a dextran polymer enhancing system (Envision™, Dako). Staining was visualized by using 3,3′-diaminobenzidine chromogen (Dako). Slides were counterstained with haematoxylin (Dako).

The percentage of labeled tumor cells and staining intensity were reported for each immunostaining. A tumor was considered as ER or PR positive when at least 10% of tumor cells displayed a nuclear staining. The HER2 IHC score was given according to the Herceptest^®^ scoring system updated according to ASCO/CAP guidelines. CK5/6 and EGFR immunostains were interpreted using criteria described by Nielsen *et al*., in order to assess the molecular subtype (Nielsen CCR2004). For all RBPs and RON IHC, an immunoreactive score (IRS) was assessed, combining the percentage of positive tumor cells and staining intensity (Friedrichs et al Cancer 1993). Staining intensity was evaluated as 0 = negative, 1 = weak, 2 = moderate and 3 = strong. The percentage of labeled cells was categorized using a five-point scale *i.e*. 0 = 0%, 1 = 1 to 10%, 2 = 11 to 50%, 3 = 51 to 80%, 4 = 81 to 100%. The IRS (from 0 to 12) was obtained by multiplying intensity and percentage scores.

Tissue cores that failed to adhere to the glass slide or that did not contain an invasive tumor component were excluded from the final analysis and reported as missing data.

### Data analysis and statistical methods

Data were summarized by frequency and percentage for categorical variables and by median and range for continuous variables. Markers were divided into low and high expression using the median expression score as cut-off. Correlations between markers and clinicopathological features were evaluated using the Mann-Whitney test for continuous variables and chi-square or fisher exact test for categorical variables. All survival times were calculated from the date of breast cancer surgery. Overall survival and metastasis free survival were estimated by Kaplan-Meier methods using the following first event definition: death from any cause for overall survival and distant recurrence (*i.e*. metastasis to any visceral or bone site) for metastasis free survival. For overall survival, patients alive at the last follow-up news were censored. For metastasis free survival, patients who never presented with metastatic disease were censored at the last follow-up news. Univariate analysis was performed using the log-rank test to identify associations with prognostic factors. All factors considered significant at the *p* < 0.05 level by this method were included in a Cox multivariate analysis to identify the major independent prognostic factors. All *p* values reported were two-sided. For all statistical tests, differences were considered significant at the 5% level. Multiple testing adjustment was done using Benjamini-Hochberg procedure (Supplementary Tables S3 and S4). Statistical analyses were performed using STATA 11.0 software.

### Bioinformatics search

61,495 human 5′UTR sequences were downloaded from the UCSC Table Browser (hg19) (http://genome.ucsc.edu/). Sequences were read into R (v 3.0.2) using the Biostrings package (Pages H, Aboyoun P, Gentleman R and DebRoy S. *Biostrings: String objects representing biological sequences, and matching algorithms*. R package version 2.32.1.) and scanned for the TAGGGW motif using the vmatchPattern function. Sequences containing that motif were then searched 5′ to the TAGGGW motif for a G4 sequence. G4 were defined as a series of at least 4 matches to the pattern 'GGG' separated by gaps with width between 1 and 7 bases (custom R script adapted from the description of QFP [29]). Results of the 'GGG' pattern matches were first reduced (using the reduce function from the IRanges package) to merge overlapping G-stretches into single blocks. Gene symbols were attributed to UCSC transcript IDs using the knownGene table (hg19) also downloaded from the UCSC Table Browser. In order to utilize functional information from Ingenuity Pathway Analysis, we exported lists of gene symbols for genes annotated in IPA as being either “breast cancer” or “migration” and merged those lists with the data from the sequence search in R.

### Cell culture condition and transfection

Experiments were performed with T47D and MDA-MB-231 human breast cancer cell lines obtained from the American Type Culture Collection, and cultured in RPMI media or DMEM supplemented with 10% foetal bovine serum (FBS) (Invitrogen), respectively. Each cell line was first amplified to generate a cell master bank. All experiments were performed from this master bank. All cell lines were tested negative for mycoplasma contamination using VenorGeM advance PCR kit (Biovalley).

Cells were transfected, using lipofectamine2000 reagent (Invitrogen), with 250 ng of *in vitro* transcribed luciferase-encoding capped and polyadenylated reporter mRNA (RON-LucF, RON Δ154 LucF and LucR ; LucF «luciferase *Firefly*», LucR «Luciferase *Renilia*») generated using mmessage mmachine kit (Ambion) according to the manufacturer's instructions.

Cells were infected using lentivirus pLenti6/V5DEST expressing GFP, hnRNP A1 or the F1 mutated variant of hnRNP A1 (cytoplasmic variant) [19] using the BLOCK-iT™ Pol II miR RNAi Expression system (Invitrogen) according to the manufacturer's protocol. Stably infected cells were selected with 10 μg/ml blasticidin.

### RNA interference (RNAi)

Small interfering RNA (siRNA) oligonucleotides against hnRNP A1 (A1-1 5′-AAGGG AGGAAAUUUUGGAGGC [10] and A1-2 5′-GCUCUUC AUUGGAGGGUUG-3′) [7]) and a control siRNA si Ctr (5′-GGUCCGGCUCCCCCAAAUG) [10] were transfected with transITX2 (Mirus) according to the manufacturer's recommendations. In brief, cells at 50% confluency were transfected twice with 100 nM siRNA in a 24 h time interval. Western blot analysis was performed 72 h posttransfection. siRNA against Ron (Smart pool L-003157-00, Dharmacon) was transfected with Lipofectamine RNAimax (Invitrogen) according to the manufacturer's recommendations. In brief, cells were reverse transfected with 50nM siRNA and recovered 72 h posttransfection.

### Western blot

Western blots were performed on whole cell and tumor lysates with standard protocols using antibodies against hnRNP A1 (clone 4B10, Abcam, dilution 1:10000), HA (clone HA.11, Eurogentec, dilution 1:1000), GFP (monoclonal, Clontech, dilution 1:1000), Ron (SC-322, Santa cruz biotechnology, dilution 1:500) and actin (polyclonal, Sigma-Aldrich, St-Louis, MO, dilution 1:20000). To validate the hnRNP A1 IHC technique, 3 breast tumors with different levels of expression were selected for cross-validation by Western blot.

### qRT-PCR (quantitative reverse transcription PCR)

Total RNA was purified from cells using TRI Reagent solution (Applied Biosystems) according to the manufacturer's instructions. Reverse transcription was done with 1 μg of total RNA using RevertAid First Strand cDNA Synthesis Kit (Fermentas) and random hexamers according to the manufacturer's instructions. Data were analysed by the threshold cycle (Ct) comparative method and normalized to the actin gene (Supplementary Figure S3A, and S3C) or the GAPDH gene (Supplementary Figure S3B). Primer sequences are the following for: Ron (Forward: 5′-GGCTGAGGTCAAGGATGTGC-3′ and reverse: 5′-GCCTGGTCTATGTATTCTCCG-3′, Actin (5′-CTGTGGCATCCACGAAACTA-3′ and reverse: 5′-AGTACTTGCGCTCAGGAGGA-3′) and GAPDH (Forward: 5′-TCAAGAAGGTGGTGAAGCAG-3′ and reverse: 5′-CCCTGTTGCTGTAGCCAAAT-3′). The primers used for *RON* amplify a region spanning exons 14 and 15 that is present in the different alternatively spliced RNA isoforms of the *RON* gene.

### Migration assays

Control and transfected cells in 500 μl of non-supplemented culture media were plated into the upper wells of 24-well transmigration chambers (ThinCert™, Greiner Bio-One, Courtaboeuf, France), and 850 μl of 10% FBS media was added into the lower wells as a chemoattractant. After a 24h-incubation at 37°C, the transmigration chambers were washed in PBS and the transmigration membranes were fixed with 10% TCA and stained with amidoschwarz. The number of migrating cells was counted on three distinct fields of each membrane (*n* = 3 for each condition).

### Cell proliferation assay

Cell proliferation assay was performed using the WST-1 reagent (Roche) according to the manufacturer's instructions.

### Immunofluorescence experiment

Cells were grown on sterilized glass slides (Dako) and were fixed in 3% paraformaldehyde for 15 min and permeabilized in 0.2% Triton for 5 min. Nonspecific binding sites were blocked with 0.1M PBS and 1% bovine serum albumin for 30 min at room temperature. Subsequently, cells were incubated for 1h with the anti-hnRNP A1 antibody (clone 4B10, Abcam), followed by 30 min incubation with an anti-mouse immunoglobulin G fluorescein isothyocyanate conjugated secondary antibody (Sigma-Aldrich).

### Polysomal fractionation analysis

Transduced T47D cells (30 millions) were treated with 0.1 mg/ml cycloheximide (CHX) for 15 min at 37°C, washed twice with ice-cold PBS/CHX, and scraped in PBS/CHX. After centrifugation for 5 min at 3000 rpm, the cell pellet was resuspended in 400 μl of LSB buffer (20 mM Tris pH 7.5, 100 mM NaCl, 3 mM MgCl_2_ and 100 U/ml RNAsine). After 13 strokes of Dounce homogeneization, 400 μl of LSB containing 0.2% Triton X-100 and 0.25 M sucrose was added. Cellular debris was removed by centrifugation and the lysate was layered on a 11.3 ml continuous sucrose gradient (15-50% sucrose in LSB buffer). After 120 min of ultracentrifugation at 38,000 rpm in a SW41-Ti rotor at 4°C, fractions were collected with an ISCO density gradient fractionation system (Foxy Jr fraction collector coupled to UA-6UV detector, Lincoln, NE). The settings were as follows: pump speed, 0.75 ml/min; fraction time, 1.2 min/fraction; chart speed, 120 cm/h; sensitivity of the OD254 recorder, 2. The absorbance at 254 nm was measured continuously as a function of gradient depth; 16 fractions of 0.9 ml were collected. The fractions recovered from the gradient were either analyzed individually or divided into two groups, fractions containing actively translated mRNAs (polysomes (P)) and fractions containing untranslated mRNAs (non-polysomes (NP)). Equal amounts of RNA from the NP and P fractions were extracted by using Trizol LS (Invitrogen), analysed by agarose gel and subjected to qRT-PCR analysis to determine the polysomal mRNA distribution.

### RNA structural-probing

*In vitro* transcribed RNA (4 pmol) was annealed with 105 cpm of ATP-labeled primer (5′-ATGTTTTTGGCGTCTTCGGC-3′), followed by reverse transcription with 5 U of AMV reverse transcriptase (Promega).

### *In vitro* translation assays in rabbit reticulocyte lysate (RRL)

100 ng of each *in vitro* transcribed luciferase reporter mRNA (RON-LucF and RON Δ154-LucF) was incubated for 30 min at 90°C in 10 μl of RRL. When indicated, (i) the reaction was performed in presence of 50 ng GST or GST-A1 recombinant protein and 100X selex hnRNP A1 primer, (ii) the *in vitro* transcribed mRNA was pre-incubated 30 min at room temperature with increasing amount of G4 ligands Phen-DC(3) and Phen-DC(6).

### Luciferase assays

For the *in vitro* translation in RRL, 5 μl from each reaction was combined with Firefly luciferase-specific substrates and light emission was measured according to manufacturer's protocol (Promega). For the analysis of the luciferase activity *in vivo*, the T47D cells transfected with the different luciferase LucF and LucR mRNAs were harvested in 100 μl of Passive Lysis Buffer (Promega). 20 μl of this extract were analyzed.

### UV cross-linking and immunoprecipitation

UV cross-linking experiments and immunoprecipitation of cross-linked hnRNP A1 with the 4B10 monoclonal antibody (mAb) were performed as described previously [7]. Protein extracts (10 μg) were mixed with *in vitro* transcribed 32P-labeled RNAs (150,000 cpm) in buffer GS (5 mM HEPES-KOH, pH 7.6, 30 mM KCl, 2 mM MgCl2, 0.2 mM dithiothreitol, and 4% glycerol) for 10 min. Reaction mixtures were irradiated on ice with UV light (254 nm) in a Stratalinker (Stratagene) at 0.4 J/cm2 at a 10-cm distance. Five units of RNAse ONE (Promega) was then added and the reaction mixtures were incubated for 45 min at 37°C. SDS gel loading buffer was added and the samples were boiled 2 min before fractionation on a 10% SDS-polyacrylamide gel. For immunoprecipitation of UV cross-linked proteins, the RNAse ONE-treated samples were diluted in 150 ml of IP buffer (50 mM Tris-HCl, pH 7.5, 150 mM NaCl, 1 mM EDTA, and 1% Triton X-100), precleared and mixed with 1 μl of anti-hnRNP A1 mAb (4B10). The mixtures were allowed to rotate 1 h at 4°C. Then, 50 μl of protein A beads was added to the mixtures and incubation continued for an additional 1 h at 4°C. After extensive washing of the beads, bound proteins were eluted in SDS-loading buffer.

## SUPPLEMENTARY FIGURES AND TABLES
















