# Supplementary material for: hnRNP A1-mediated translational regulation of the G quadruplex-containing RON receptor tyrosine kinase mRNA linked to tumor progression
Source: Oncotarget. 2016 Feb 22;7(13):16793–805. doi: 10.18632/oncotarget.7589 (PMC4941351; doi:10.18632/oncotarget.7589)
Supplement: Supplementary file 1 [file oncotarget-07-16793-s001.pdf]

SUPPLEMENTARY FIGURES AND TABLES

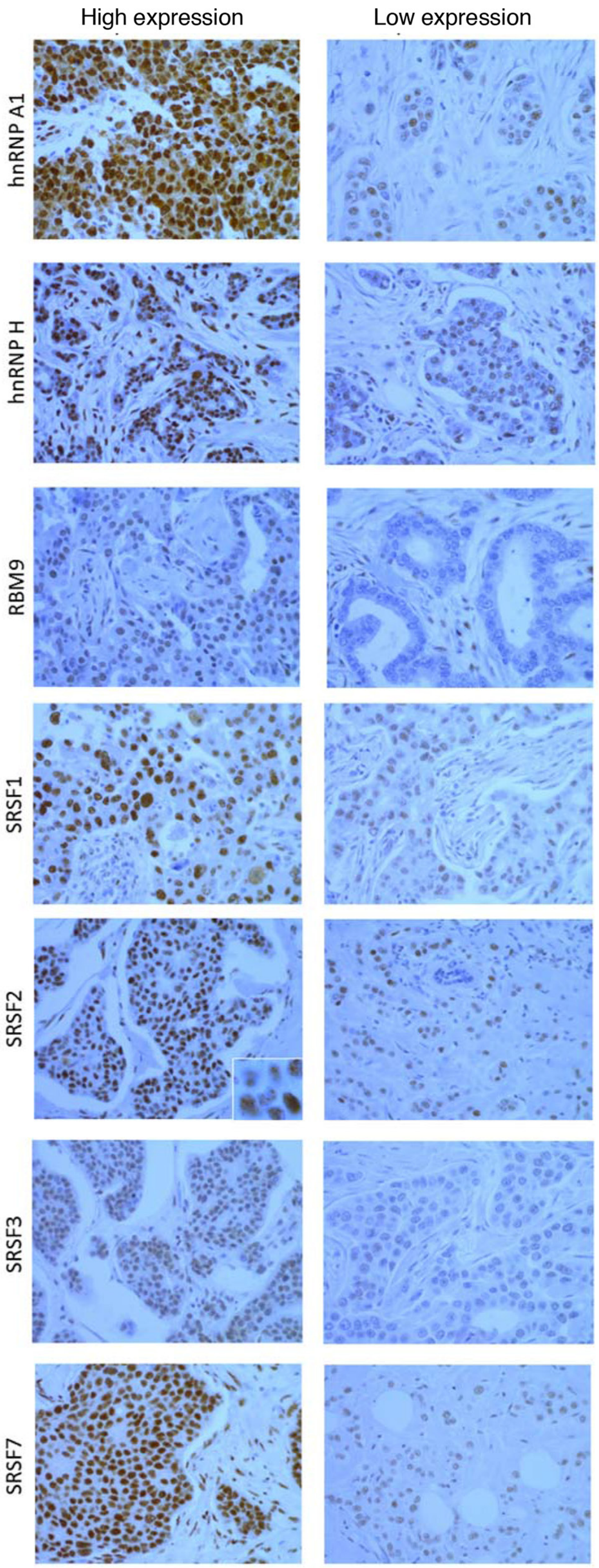

Supplementary Figure S1: Representative examples of immunohistochemistry in a low expressing or a high expressing tumor for each of the proteins indicated on the left.

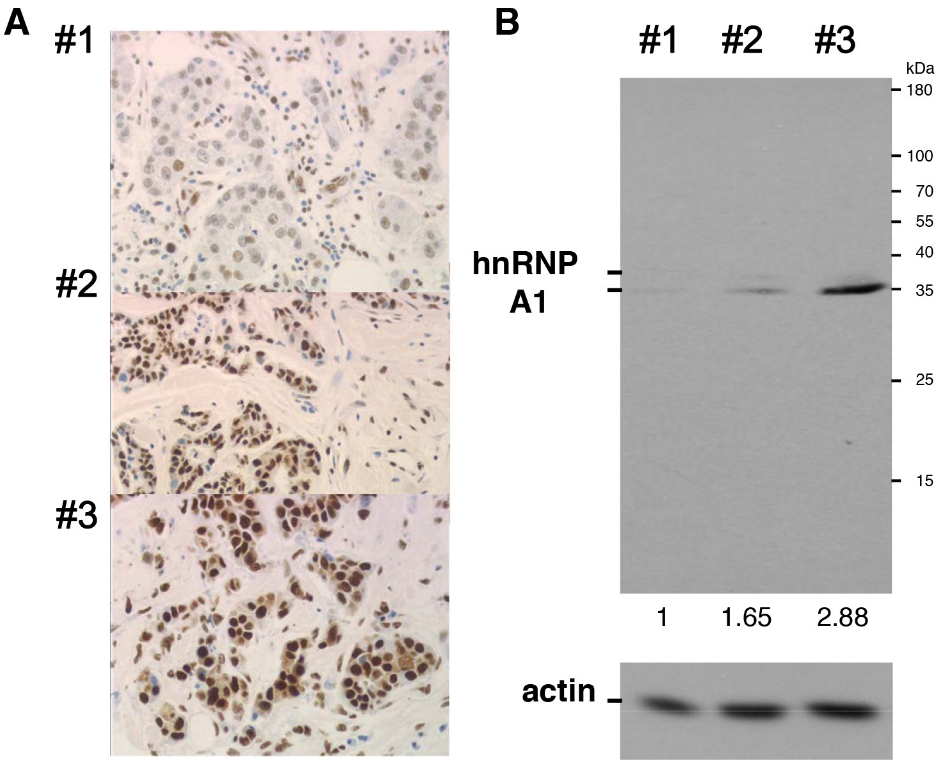

Supplementary Figure S2: A. Immunohistochemistry (with the 4B10 antibody against hnRNP A1) performed in three representative breast tumor samples. **B.** Western blot analysis of hnRNP A1 (4B10 antibody) in the same three tumor samples as in A.

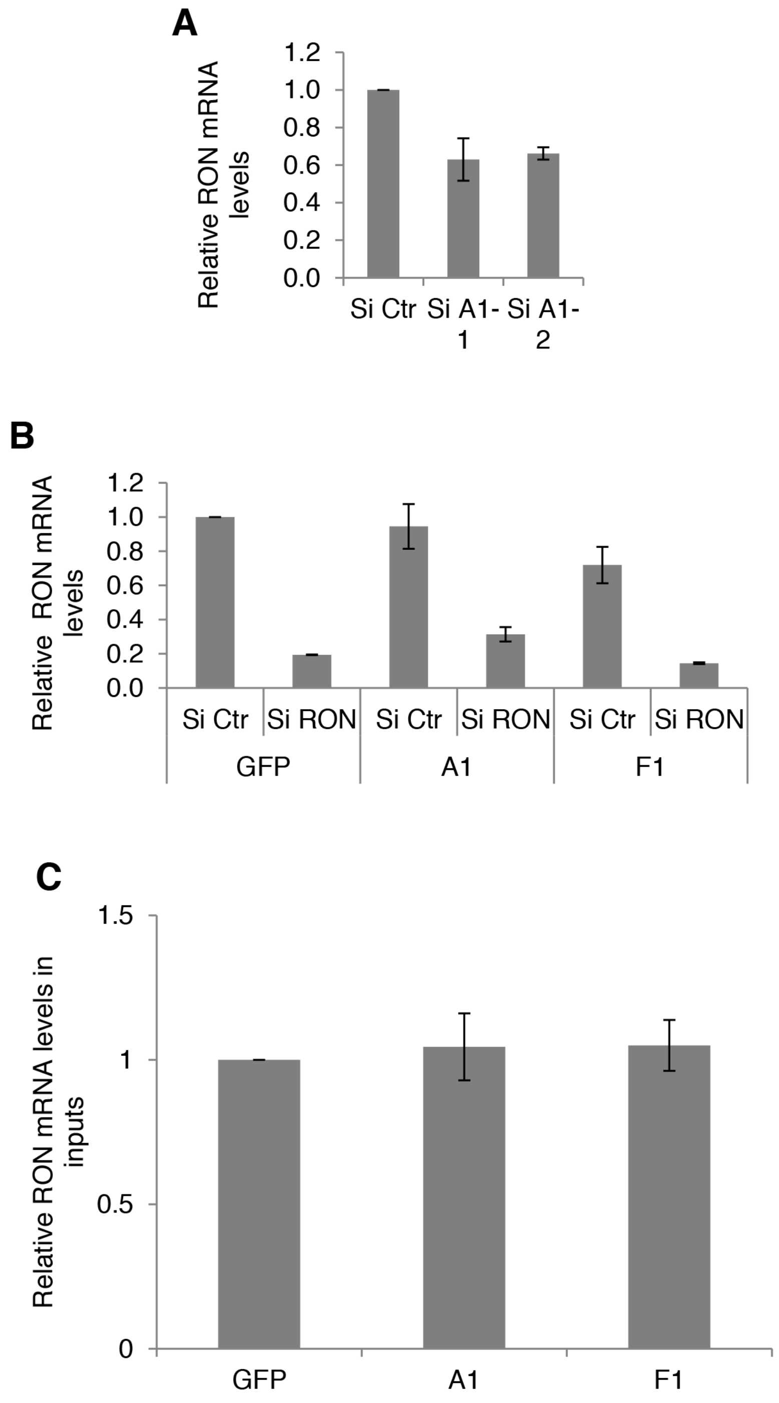

Supplementary Figure S3: Expression of *RON* mRNA (as determined by qRT-PCR) in the indicated conditions corresponding to Figure 3B (A), Figure 3C (B) and Figure 4 (C).

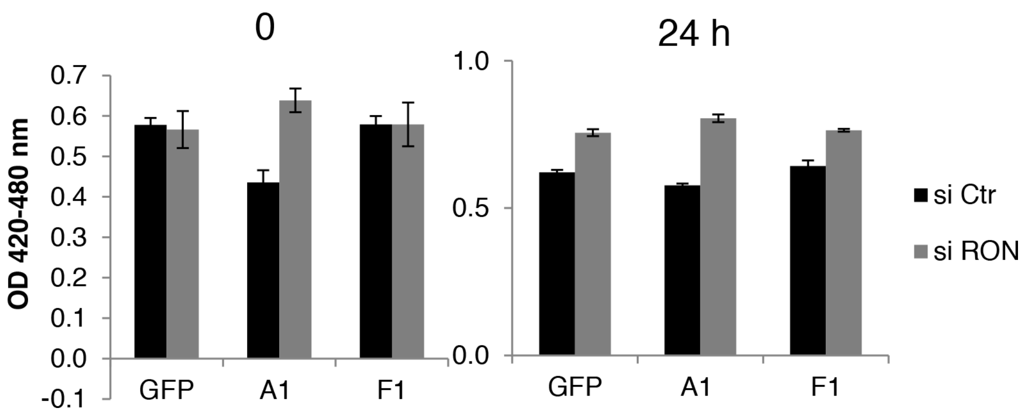

Supplementary Figure S4: Cell proliferation assay. The analysis was done at the beginning of the experiment (O) and 24 h later (24 h).

Supplementary Table S1: Patient and tumour characteristics.

(See Supplementary File 1)

Supplementary Table S2: Distribution of RBP expression in breast cancers (n=277; collection 1).

(See Supplementary File 2)

Supplementary Table S3: Correlation between RBP expression and histopathological features (collection 1).

(See Supplementary File 3)

Supplementary Table S4: Correlation between RBP expression and metastasis-free survival in breast cancer (n=277) (univariate analysis).

(See Supplementary File 4)

Supplementary Table S5: Characteristics of tumours displaying an associated cytoplasmic localization of hnRNP A1 (n=14 out of 254 invasive breast carcinomas; collection 1).

(See Supplementary File 5)

Supplementary Table S6: Characteristics of tumours displaying low vs high expression of hnRNP A1 in 254 invasive breast carcinomas.

(See Supplementary File 6)

Supplementary Table S7: List of 120 5'UTRs from cell migration and breast cancer encoding mRNAs that contain at least one UAGGGA/U sequence. The number of G4 predicted sequence and the length of the 5'UTR are indicated.

(See Supplementary File 7)
