# Supplementary material for: hnRNP A1-mediated translational regulation of the G quadruplex-containing RON receptor tyrosine kinase mRNA linked to tumor progression
Source: Oncotarget. 2016 Feb 22;7(13):16793–805. doi: 10.18632/oncotarget.7589 (PMC4941351; doi:10.18632/oncotarget.7589)
Supplement: Supplementary file 2 [file oncotarget-07-16793-s002.docx]

**Table S1. Patient and tumour characteristics**

| **Characteristics** | **Collection 1 n=277** | **Collection 2**  **n=113** |
| --- | --- | --- |
| **Age** |  |  |
| median (range) | 54 yr (29 - 87) | 60 yr (48 - 74) |
| **Tumour size** |  |  |
| T<20mm | 130 (50.6%) | 37 (35.2%) |
| T≥20mm | 127 (49.4%) | 68 (64.8%) |
| NA | 20 | 8 |
| median (range) | 18 mm (4 – 120) | 20 mm (4 - 200) |
| **Histological type** |  |  |
| Ductal of no special type | 214 (79%) | 91 (80.5%) |
| lobular | 47 (17.3%) | 13 (11.5%) |
| Others  NA | 10 (3.7%)  6 | 9 (8%)  0 |
| **Histological grade** |  |  |
| I | 34 (12.7%) | 18 (16.2%) |
| II | 108 (40.1%) | 47 (42.3%) |
| III | 127 (47.2%) | 46 (41.5%) |
| NA | 8 | 2 |
| **Auxillary node status** |  |  |
| - | 157 (56.7%) | 39 (34.8%) |
| + | 120 (43.3%) | 73 (65.2%) |
| NA | 0 | 1 |
| **ER** |  |  |
| + | 180 (68%) | 74 (66.1%) |
| - | 85 (32%) | 38 (33.9%) |
| NA | 12 | 1 |
| **PR** |  |  |
| + | 162 (60.7%) | 59 ( 52.7%) |
| - | 105 (39.3%) | 53 ( 47.3%) |
| NA | 10 | 1 |
| **HER2 (IHC)** |  |  |
| + (overexpression) | 33 (12.7%) | 9 (8%) |
| - | 226 (87.3%) | 103 (92%) |
| NA | 18 | 1 |
| **Molecular subtype***  Luminal  **HER2**  **basal-like**  **triple-negative non basal**  **NA** | 186 (70.7%)  33 (12.6%)  30 (11.4%)  14 (5.3%)  14 | 79 (70.5%)  9 (8.1%)  8 (7.1%)  16 (14.3%)  1 |
| **Relapse** |  |  |
| no | 176 (64.5%) | 38 (33.6%) |
| Yes  **NA** | 97 (35.5%)  4 | 75 (66.4%)  0 |
| **Type of relapse** |  |  |
| loco-regional | 29 (30%) | 12 (16%) |
| distant metastasis | 68 (70%) | 63 (84%) |
| **Patients deceased** |  |  |
| no | 213 (78.3%) | 39 (34.5%) |
| Yes  **NA** | 59 (21.7%)  5 | 74 (65.5%)  0 |
| **Total follow-up** |  |  |
| median | 104 months | 242.9 months |
| CI 95% | 95 - 109 | 220 - 254 |

* molecular subtype according to IHC surrogate as described by Nielsen *et al*. CI: confidence interval; ER: oestrogen receptor; IHC, Immunohistochemistry; NA, Not Available; PR: progesterone receptor.
