# Supplementary material for: hnRNP A1-mediated translational regulation of the G quadruplex-containing RON receptor tyrosine kinase mRNA linked to tumor progression
Source: Oncotarget. 2016 Feb 22;7(13):16793–805. doi: 10.18632/oncotarget.7589 (PMC4941351; doi:10.18632/oncotarget.7589)
Supplement: Supplementary file 3 [file oncotarget-07-16793-s003.docx]

Table S2. Distribution of RBP expression in breast cancers (n=277; collection 1)

|  | **hnRNP A1** | **hnRNP H** | **RBM9** | **SRSF1** | **SRSF2** | **SRSF3** | **SRSF7** |
| --- | --- | --- | --- | --- | --- | --- | --- |
| **Interpretable case** (n) | 254 | 250 | 251 | 252 | 256 | 249 | 255 |
| **% of stained cells**  median (range) | 90 (0-100) | 80 (5-100) | 0 (0-90) | 80 (0-100) | 70 (0-100) | 70 (0-100) | 80 (5-100) |
| **Staining intensity** |  |  |  |  |  |  |  |
| 0 | 1 (0.4%) | 0 (0%) | 142 (56.6%) | 4 (1.6%) | 6 (2.3%) | 30 (12%) | 0 (0%) |
| 1+ | 11 (4.3%) | 60 (24%) | 100 (39.8%) | 97 (38.5%) | 86 (33.6%) | 110 (44.2%) | 98 (38.4%) |
| 2+ | 64 (25.2%) | 95 (38%) | 9 (3.6%) | 93 (36.9%) | 68 (26.6%) | 63 (25.3%) | 99 (38.8%) |
| 3+ | 178 (70.1%) | 95 (38%) | 0 (0%) | 58 (23%) | 96 (37.5%) | 46 (18.5%) | 58 (22.7%) |
| **IRS**  median (range) | 12 (0-12) | 8 (1-12) | 0 (0-6) | 6 (0-12) | 6 (0-12) | 3 (0-12) | 6 (1-12) |

IRS : immunoreactive score
