# Supplementary material for: hnRNP A1-mediated translational regulation of the G quadruplex-containing RON receptor tyrosine kinase mRNA linked to tumor progression
Source: Oncotarget. 2016 Feb 22;7(13):16793–805. doi: 10.18632/oncotarget.7589 (PMC4941351; doi:10.18632/oncotarget.7589)
Supplement: Supplementary file 4 [file oncotarget-07-16793-s004.docx]

Table S3. Correlation between RBP expression and histopathological features (collection 1)

|  | **Histological grade** | | | |  | **Lymph node** | | |
| --- | --- | --- | --- | --- | --- | --- | --- | --- |
|  | **I**  **(n=30)** | **II**  **(n=101)** | **III**  **(n=122)** | ***p*** |  | **N-**  **(n=143)** | **N+**  **(n=118)** | ***p*** |
| **hnRNP A1**  median IRS | 9 (2-12) | 12 (3-12) | 12 (0-12) | 0.454 |  | 9 (0-12) | 12 (1-12) | **<0.001** |
| **hnRNP H**  median IRS | 6 (1-12) | 8 (1-12) | 8 (1-12) | **0.048** |  | 8 (1-12) | 6 (1-12) | **0.003** |
| **RBM9**  median IRS | 0 (0-2) | 0 (0-6) | 1 (0-6) | 0.103 |  | 0 (0-6) | 0 (0-6) | 0.352 |
| **SRSF1**  median IRS | 6 (1-12) | 6 (0-12) | 6 (0-12) | 0.488 |  | 6 (0-12) | 8 (0-12) | 0.466 |
| **SRSF2**  median IRS | 6 (1-12) | 6 (0-12) | 6 (0-12) | 0.223 |  | 6 (0-12) | 6 (0-12) | 0.392 |
| **SRSF3**  median IRS | 4 (0-12) | 4 (0-12) | 3 (0-12) | **0.030** |  | 6 (0-12) | 2 (0-12) | **<0.001** |
| **SRSF7**  median IRS | 5 (1-12) | 6 (1-12) | 6 (1-12) | 0.074 |  | 6 (1-12) | 8 (1-12) | **<0.001** |

|  | **Estrogen receptor** | | |  | **HER2** | | |
| --- | --- | --- | --- | --- | --- | --- | --- |
|  | **ER+**  **(n=171)** | **ER-**  **(n=82)** | ***p*** |  | **HER2-**  **(n=223)** | **HER2+**  **(n=33)** | ***p*** |
| **hnRNP A1**  median IRS | 12 (2-12) | 10.5 (0-12) | 0.213 |  | 12 (0-12) | 12 (2-12) | 0.124 |
| **hnRNP H**  median IRS | 8 (1-12) | 8 (1-12) | 0.545 |  | 8 (1-12) | 6 (2-12) | 0.288 |
| **RBM9**  median IRS | 0 (0-6) | 1 (0-6) | **<0.001** |  | 0 (0-6) | 1 (0-3) | 0.085 |
| **SRSF1**  median IRS | 6 (0-12) | 6 (0-12) | 0.325 |  | 6 (0-12) | 8 (1-12) | 0.487 |
| **SRSF2**  median IRS | 6 (0-12) | 6 (0-12) | 0.791 |  | 6 (0-12) | 8 (1-12) | 0.267 |
| **SRSF3**  median IRS | 4 (0-12) | 3 (0-12) | **<0.001** |  | 4 (0-12) | 2 (0-12) | **0.003** |
| **SRSF7**  median IRS | 6 (1-12) | 6 (2-12) | 0.799 |  | 6 (1-12) | 8 (2-12) | **0.017** |

ER : estrogen receptor ; IRS : immunoreactive score

Table S3. Correlation between RBP expression and histopathological features

|  | **Histological grade** | | | |  |  | **Lymph node** | | |  |
| --- | --- | --- | --- | --- | --- | --- | --- | --- | --- | --- |
|  | **I**  **(n=30)** | **II**  **(n=101)** | **III**  **(n=122)** | ***p*** | ***p adjusted*** |  | **N-**  **(n=143)** | **N+**  **(n=118)** | ***p*** | ***p adjusted*** |
| **hnRNP A1**  median IRS | 9 (2-12) | 12 (3-12) | 12 (0-12) | 0.454 | 0.488 |  | 9 (0-12) | 12 (1-12) | **<0.001** | **0.002** |
| **hnRNP H**  median IRS | 6 (1-12) | 8 (1-12) | 8 (1-12) | **0.048** | 0.168 |  | 8 (1-12) | 6 (1-12) | **0.003** | **0.005** |
| **RBM9**  median IRS | 0 (0-2) | 0 (0-6) | 1 (0-6) | 0.103 | 0.180 |  | 0 (0-6) | 0 (0-6) | 0.352 | 0.457 |
| **SRSF1**  median IRS | 6 (1-12) | 6 (0-12) | 6 (0-12) | 0.488 | 0.488 |  | 6 (0-12) | 8 (0-12) | 0.466 | 0.466 |
| **SRSF2**  median IRS | 6 (1-12) | 6 (0-12) | 6 (0-12) | 0.223 | 0.312 |  | 6 (0-12) | 6 (0-12) | 0.392 | 0.457 |
| **SRSF3**  median IRS | 4 (0-12) | 4 (0-12) | 3 (0-12) | **0.030** | 0.168 |  | 6 (0-12) | 2 (0-12) | **<0.001** | **0.002** |
| **SRSF7**  median IRS | 5 (1-12) | 6 (1-12) | 6 (1-12) | 0.074 | 0.173 |  | 6 (1-12) | 8 (1-12) | **<0.001** | **0.002** |

|  | **Estrogen receptor** | | |  |  | **HER2** | | |  |
| --- | --- | --- | --- | --- | --- | --- | --- | --- | --- |
|  | **ER+**  **(n=171)** | **ER-**  **(n=82)** | ***p*** | ***p adjusted*** |  | **HER2-**  **(n=223)** | **HER2+**  **(n=33)** | ***p*** | ***p* adjusted** |
| **hnRNP A1**  median IRS | 12 (2-12) | 10.5 (0-12) | 0.213 | 0.497 |  | 12 (0-12) | 12 (2-12) | 0.124 | 0.217 |
| **hnRNP H**  median IRS | 8 (1-12) | 8 (1-12) | 0.545 | 0.763 |  | 8 (1-12) | 6 (2-12) | 0.288 | 0.336 |
| **RBM9**  median IRS | 0 (0-6) | 1 (0-6) | **<0.001** | **0.004** |  | 0 (0-6) | 1 (0-3) | 0.085 | 0.198 |
| **SRSF1**  median IRS | 6 (0-12) | 6 (0-12) | 0.325 | 0.569 |  | 6 (0-12) | 8 (1-12) | 0.487 | 0.487 |
| **SRSF2**  median IRS | 6 (0-12) | 6 (0-12) | 0.791 | 0.799 |  | 6 (0-12) | 8 (1-12) | 0.267 | 0.336 |
| **SRSF3**  median IRS | 4 (0-12) | 3 (0-12) | **<0.001** | **0.004** |  | 4 (0-12) | 2 (0-12) | **0.003** | **0.021** |
| **SRSF7**  median IRS | 6 (1-12) | 6 (2-12) | 0.799 | 0.799 |  | 6 (1-12) | 8 (2-12) | **0.017** | **0.060** |

ER : estrogen receptor ; IRS : immunoreactive score
