# Supplementary material for: hnRNP A1-mediated translational regulation of the G quadruplex-containing RON receptor tyrosine kinase mRNA linked to tumor progression
Source: Oncotarget. 2016 Feb 22;7(13):16793–805. doi: 10.18632/oncotarget.7589 (PMC4941351; doi:10.18632/oncotarget.7589)
Supplement: Supplementary file 5 [file oncotarget-07-16793-s005.docx]

Table S4. Correlation between RBP expression and metastasis-free survival in breast cancer (n=277) (univariate analysis)

|  | **Event / N** | **Survival**  **(10 years)** | **HR** | **CI95%** | ***p*** | ***P adjusted*** |
| --- | --- | --- | --- | --- | --- | --- |
| **hnRNP A1 (IRS)**  Low expression  High expression | 22 / 109  43 / 145 | 74.8%  60.6% | 1.00  1.73 | 1.03-2.90 | **0.036** | 0.144 |
| **hnRNP H (IRS)**  Low expression  High expression | 33 / 112  33 / 138 | 51.2%  74.1% | 1.00  0.76 | 0.47-1.23 | 0.262 | 0.574 |
| **RBM9 (IRS)**  Low expression  High expression | 40 / 142  26 / 109 | 64.1%  69.1% | 1.00  0.91 | 0.55-1.49 | 0.708 | 0.797 |
| **SRSF1 (IRS)**  Low expression  High expression | 33 / 143  31 / 109 | 68.8%  66.4% | 1.00  1.31 | 0.80-2.14 | 0.287 | 0.574 |
| **SRSF2 (IRS)**  Low expression  High expression | 40 / 146  26 / 110 | 66%  68.8% | 1.00  0.87 | 0.53-1.43 | 0.590 | 0.786 |
| **SRSF3 (IRS)**  Low expression  High expression | 35 / 128  29 / 121 | 63.5%  70.5% | 1.00  0.87 | 0.53-1.43 | 0.583 | 0.786 |
| **SRSF7 (IRS)**  Low expression  High expression | 39 / 152  27 / 103 | 66.2%  68.1% | 1.00  1.07 | 0.65-1.74 | 0.797 | 0.797 |
| **hnRNP A1**  **(Cytoplasmic localization)**  No  Yes | 57 / 240  8/ 14 | 24.5  70.1 | 1.00  3.43 | 1.63-7.21 | **0.0005** | **0.004** |

CI: confidence interval; HR: hazard ratio; IRS: immunoreactive score.
