# Supplementary material for: hnRNP A1-mediated translational regulation of the G quadruplex-containing RON receptor tyrosine kinase mRNA linked to tumor progression
Source: Oncotarget. 2016 Feb 22;7(13):16793–805. doi: 10.18632/oncotarget.7589 (PMC4941351; doi:10.18632/oncotarget.7589)
Supplement: Supplementary file 6 [file oncotarget-07-16793-s006.docx]

**Table S5. Characteristics of tumours displaying an associated cytoplasmic localization of hnRNP A1 (n=14 out of 254 invasive breast carcinomas; collection 1)**

| **Characteristics** | **Cytoplasmic localization**  **n=14** | **No cytoplasmic localization**  **n=240** | **p value** |
| --- | --- | --- | --- |
| **HnRNP A1** |  |  | p=0.0260 |
| low expression | 2 (14.3%) | 107 (44.6%) |  |
| high expression | 12 (85.7%) | 133 (55.4%) |  |
| **Age** |  |  | p=0.6332 |
| median (range) | 52 yr (30 – 87) | 53 yr (29 - 83) |  |
| **Tumour size** |  |  | p=0.0260 |
| median  (range) | 27mm  (10 – 50) | 19 mm (4 - 120) |  |
| NA | 1 | 19 |  |
| **Histological type** |  |  | p=0.1028 |
| ductal NST | 10 (76.9%) | 190 (80.8%) |  |
| lobular | 1 (7.7%) | 38 (16.2%) |  |
| others | 2 (15.4%) | 7 (3%) |  |
| NA | 1 | 5 |  |
| **Histological grade** |  |  | p=0.2771 |
| I | 0 | 29 (12.4%) |  |
| II | 4 (30.8%) | 94 (40.2%) |  |
| III | 9 (69.2%) | 111 (47.4%) |  |
| NA | 1 | 6 |  |
| **Auxiliary node status** |  |  | p=0.0014 |
| - | 2 (14.3%) | 139 (57.9%) |  |
| + | 12 (85.7%) | 101 (42.1%) |  |
| **ER status** |  |  | p=0.3568 |
| + | 7 (53.8%) | 162 (69.2%) |  |
| - | 6 (46.2%) | 72 (30.8%) |  |
| NA | 1 | 6 |  |
| **PR status** |  |  | p=0.2446 |
| + | 6 (46.2%) | 147 (62.3%) |  |
| - | 7 (53.8%) | 89 (37.7%) |  |
| NA | 1 | 4 |  |
| **HER2 (IHC)** |  |  | p=1.000 |
| + | 1 (7.7%) | 30 (12.7%) |  |
| - | 12 (92.3%) | 206 (87.3%) |  |
| NA | 1 | 4 |  |
| **Molecular subtype*** |  |  | p=0.3498 |
| luminal | 8 (61.5%) | 169 (71.6%) |  |
| HER2 | 1 (7.7%) | 30 (12.7%) |  |
| basal-like | 3 (23.1%) | 25 (10.6%) |  |
| triple-negative non basal | 1 (7.7%) | 12 (5.1%) |  |
| NA | 1 | 4 |  |
|  |  |  |  |
| **Metastatic relapse** |  |  | p=0.0005 |
| number of events | 8 (57%) | 57 (23.7%) |  |
| MFS rate (10-yr) | 24.5% | 70.1% |  |
| **Overall survival** |  |  | p=0.0087 |
| patients deceased | 6 (42.8%) | 50 (22%) |  |
| OS rate (10-yr) | 31.2% | 70.1% |  |

*molecular subtype according to IHC surrogate as described by Nielsen *et al*. ER: estrogen receptor; IHC, Immunohistochemistry; MFS: metastasis free survival; NA, Not Available; NST: no special type; OS: overall survival; PR: progesterone receptor.
