# Supplementary material for: hnRNP A1-mediated translational regulation of the G quadruplex-containing RON receptor tyrosine kinase mRNA linked to tumor progression
Source: Oncotarget. 2016 Feb 22;7(13):16793–805. doi: 10.18632/oncotarget.7589 (PMC4941351; doi:10.18632/oncotarget.7589)
Supplement: Supplementary file 7 [file oncotarget-07-16793-s007.docx]

**Table S6. Characteristics of tumours displaying low vs high expression of hnRNP A1 in 254 invasive breast carcinomas**

| **Characteristics** | **n=254** | | |
| --- | --- | --- | --- |
|  | **Low expression**  **n=109** | **High expression**  **n=145** | ***p*** |
| **Age** |  |  | p=0.0857 |
| median (range) | 54 yr (30 - 83) | 52 yr (29 - 87) |  |
| **Tumour size** |  |  | p=0.4882 |
| median (range) | 18 mm (8 - 70) | 20 mm (4 - 120) |  |
| NA | 4 | 16 |  |
| **Histological type** |  |  | p =0.6377 |
| ductal NST | 89 (81.7%) | 111 (79.9%) |  |
| lobular | 18 (16.5%) | 21 (15.1%) |  |
| others | 2 (1.8%) | 7 (5%) |  |
| NA | 0 | 6 |  |
| **Histological grade** |  |  | p =0.6183 |
| I | 15 (13.9%) | 14 (10%) |  |
| II | 43 (39.8%) | 55 (39.6%) |  |
| III | 50 (46.3%) | 70 (50.4%) |  |
| NA | 1 | 6 |  |
| **Auxiliary node status** |  |  | p <0.0001 |
| - | 89 (81.7%) | 52 (35.9%) |  |
| + | 20 (18.3%) | 93 (64.1%) |  |
| NA | 0 | 0 |  |
| **ER status** |  |  | p =0.2068 |
| + | 70 (64.2%) | 99 (71.7%) |  |
| - | 39 (35.8%) | 39 (28.3%) |  |
| NA | 0 | 7 |  |
| **PR status** |  |  | p =0.6041 |
| + | 65 (59.6%) | 88 (62.9%) |  |
| - | 44 (40.4%) | 52 (37.1%) |  |
| NA | 0 | 5 |  |
| **HER2 (IHC)** |  |  | p =0.0770 |
| + | 9 (8.3%) | 22 (15.7%) |  |
| - | 100 (91.7%) | 118 (84.3%) |  |
| NA | 0 | 5 |  |
| **Classification** |  |  | p =0.2384 |
| HR+/HER2- | 78 (71.5%) | 97 (70.8%) |  |
| HR+/HER2+ | 4 (3.7%) | 7 (5.1%) |  |
| HR-/HER2+ | 5 (4.6%) | 14 (10.2%) |  |
| HR-/HER2- | 22 (20.2%) | 19 (13.9%) |  |
| NA | 0 | 8 |  |
| **Molecular subtype*** |  |  | p =0.2241 |
| luminal | 78 (71.6%) | 99 (70.7%) |  |
| HER2 | 9 (8.3%) | 22 (15.7%) |  |
| basal-like | 15 (13.8%) | 13 (9.3%) |  |
| triple-negative non basal | 7 (6.3%) | 6 (4.3%) |  |
| NA | 0 | 5 |  |

* molecular subtype according to IHC surrogate as described by Nielsen *et al*. ER: estrogen receptor; HR : hormone receptor ; IHC, Immunohistochemistry; NA, Not Available; NST: no special type; PR: progesterone receptor.
