# Supplementary material for: hnRNP A1-mediated translational regulation of the G quadruplex-containing RON receptor tyrosine kinase mRNA linked to tumor progression
Source: Oncotarget. 2016 Feb 22;7(13):16793–805. doi: 10.18632/oncotarget.7589 (PMC4941351; doi:10.18632/oncotarget.7589)
Supplement: Supplementary file 8 [file oncotarget-07-16793-s008.docx]

**Table S7.** List of 120 5'UTRs from cell migration and breast cancer encoding mRNAs that contain at least one UAGGGA/U sequence. The number of G4 predicted sequence and the length of the 5’UTR are indicated.

A Number of TAGGGW

B Number of predicted G4

C 5'UTR length

UCID SYMBOL A B C

uc001fgq.1 ADAM15 5 10 2591

uc002egr.1 CYLD 1 6 302

uc001pvk.3 DPAGT1 2 4 1145

uc009zlf.4 TUBA1A 2 3 905

uc001xgx.3 ESR2 1 2 1728

uc003lzg.3 HMMR 1 1 182

uc011kux.2 KCNH2 1 1 1121

uc011bdd.2 MST1R 2 1 264

uc001cjw.3 PTPRF 1 1 822

uc009wgk.1 RHOC 1 1 1486

uc002yuo.1 RUNX1 1 1 1578

uc002ojk.3 HNRNPL 1 3 1342

uc010haz.2 MAPK11 1 2 527

uc003jfu.2 MYO10 1 2 468

uc001rql.3 VDR 1 2 401

uc001bol.3 FGR 1 1 212

uc002qto.3 MZF1 1 1 561

uc001hln.3 TGFB2 2 1 1368

uc001rss.3 WNT10B 1 1 444

uc003hyg.3 AIMP1 1 0 674

uc001ypl.3 AKT1 2 0 958

uc002one.3 AKT2 1 0 640

uc003xxp.1 ARFGEF1 1 0 286

uc001gtw.4 ASPM 1 0 672

uc010kch.3 BACH2 1 0 1011

uc001rge.4 BCAT1 1 0 527

uc011asw.2 BHLHE40 1 0 996

uc003kpg.3 CAMK4 1 0 936

uc003eew.4 CASR 1 0 438

uc003vig.2 CAV1 1 0 860

uc010yoz.1 CCDC88A 1 0 842

uc010sgv.1 CD69 1 0 81

uc002wjl.3 CDC25B 1 0 277

uc002ewf.2 CDH3 1 0 1132

uc003cfv.3 CLASP2 1 0 160

uc003isi.3 CLCN3 1 0 559

uc010cmc.3 CLDN7 2 0 902

uc001rmm.2 CNTN1 1 0 227

uc002iqm.3 COL1A1 1 0 126

uc002htq.3 CSF3 1 0 302

uc010hia.1 CTNNB1 1 0 156

uc003wul.3 CTSB 1 0 294

uc001hdu.3 CTSE 1 0 118

uc010acy.1 CYSLTR2 1 0 660

uc011mkr.2 DDX3X 1 0 855

uc001ydw.2 DICER1 1 0 212

uc009yaq.3 DOCK1 1 0 1212

uc003xfa.3 DPYSL2 1 0 133

uc010nbp.3 EGFL7 1 0 534

uc003ymt.3 EIF3E 1 0 779

uc010cwa.3 ERBB2 1 0 526

uc002wmr.3 FERMT1 1 0 789

uc003lmp.5 FGF1 1 0 573

uc021pzx.1 FGFR2 1 0 319

uc002vez.3 FN1 2 0 5117

uc010kdj.1 FOXO3 1 0 232

uc009wqk.2 GBA 1 0 571

uc002xzw.3 GNAS 1 0 285

uc004dfq.4 GPR34 1 0 208

uc010ohd.1 HDAC1 1 0 63

uc003mdd.2 HRH2 1 0 1773

uc011adv.2 IFNAR1 1 0 341

uc002vgj.4 IGFBP5 1 0 774

uc022auj.1 IKBKB 1 0 399

uc001prw.3 IL10RA 3 0 2025

uc003hhe.3 IL8 1 0 153

uc001osg.3 INPPL1 1 0 853

uc010mgy.2 KANK1 1 0 2385

uc009xrt.1 KCNMA1 1 0 677

uc003dxb.4 KIAA1524 2 0 269

uc002jgl.3 KPNA2 1 0 361

uc009zmm.2 KRT8 1 0 457

uc001fnf.1 LMNA 1 0 641

uc010thv.2 LMO7 2 0 1260

uc001tbm.3 LUM 1 0 389

uc001hxi.3 LYST 1 0 3318

uc003hps.3 MAPK10 1 0 686

uc002ktp.3 MIB1 1 0 1060

uc010hkx.2 MST1 1 0 340

uc010pfi.2 MUC1 1 0 406

uc003ysi.3 MYC 1 0 525

uc002dea.1 MYH11 2 0 1304

uc002xav.3 NCOA6 2 0 2571

uc010joz.2 NEDD9 1 0 362

uc001icv.3 NLRP3 1 0 742

uc011eqx.2 NOTCH4 4 0 2488

uc001rzs.3 NR4A1 1 0 319

uc004any.1 NTRK2 1 0 938

uc002scs.4 PELI1 2 0 4039

uc001hax.1 PIK3C2B 1 0 453

uc011dtw.2 PIM1 1 0 125

uc001cyj.2 PPAP2B 1 0 568

uc003bwu.3 PPARG 1 0 274

uc002pfj.3 PRKD2 1 0 757

uc010ppg.2 PTPRC 2 0 213

uc031qjr.1 PXN 1 0 476

uc010wfe.2 RARA 1 0 590

uc002san.1 REL 1 0 224

uc001gsg.3 RGS18 1 0 176

uc010muz.2 RGS3 1 0 353

uc001gcl.4 RGS4 1 0 329

uc001loh.3 RIC8A 1 0 227

uc003dqf.1 ROBO1 1 0 788

uc031rrk.1 SERPINE2 1 0 661

uc003tfo.4 SFRP4 1 0 386

uc010uez.1 SHC4 1 0 220

uc031rtt.1 SLC12A5 1 0 677

uc010ofk.3 SLC9A1 1 0 1352

uc002lfb.4 SMAD4 1 0 2466

uc003xqp.3 SNAI2 1 0 175

uc011cdc.2 SPARCL1 1 0 473

uc001vli.3 SPRY2 1 0 978

uc002xgy.3 SRC 1 0 348

uc010ugm.1 TCF12 1 0 88

uc010wty.2 TIMP2 1 0 293

uc003pzx.1 TPD52L1 1 0 327

uc022ait.1 TRIP6 1 0 721

uc001qnn.1 VWF 1 0 250

uc002sch.3 WDPCP 1 0 462

uc001qjl.3 WNT5
